# Supplementary material for: MEMO1 binds iron and modulates iron homeostasis in cancer cells
Source: eLife. 2024 Apr 19;13:e86354. doi: 10.7554/eLife.86354 (PMC11081632; doi:10.7554/eLife.86354)
Supplement: Supplementary file 1. — (A) Iron-related genes exhibiting gene-of-function (GOF) interactions with MEMO1. (B) Iron-related genes exhibiting loss-of-function (LOF) interactions with MEMO1. (C) Genes involved in ferroptosis and exhibiting GOF or LOF interactions (highlighted in light blue) with MEMO1. (D) Structure determination statistics for MEMO1-metal complexes and the C244S mutant. [file elife-86354-supp1.docx]

**Supplementary file 1A**

**Iron-related genes exhibiting GOF interactions with *MEMO1***

| **Gene** | **GO Slim** | **P-value** | **Distance low-high** | **Protein name** | **Database** | **Protein function** |
| --- | --- | --- | --- | --- | --- | --- |
| *TFR2* | Ion regulation | 0.032 | 0.06 | Transferrin receptor 2 | CERES breast | Iron transport |
| *FTH1* | Immune System process | 0.037 | 0.19 | Ferritin H1 | DRIVE -RSA, pan-cancer | Cellular iron storage |
| *HSPA9* | Chaperone and Protein folding | 0.007 | 0.77 | Mitochondrial HSP70, Grp75, mortalin | Marcotte et al, pan-cancer | Iron-sulfur cluster biogenesis |
| *ACO1* | Metabolism | 0.019 | 0.16 | Apo-form: Iron-response protein (IRP); holo-form: Aconitase | DRIVE -Ataris, pan-cancer | Iron binding protein, aconitase/IRP |
| *ACO2* | Metabolism | 0.038 | 0.32 | Mitochondrial aconitase | CERES, pan-cancer | Fe_4_S_4_-cluster protein, TCA cycle |
|  |  | 0.032 | 0.66 |  | DRIVE -Ataris,  pan-cancer |  |
| *HMOX1* | DDR pathway & NA metabolism | 0.043 | 0.51 | Heme oxygenase 1 | DRIVE -RSA,  Breast | First enzyme of heme catabolism |
| *OGDH* | Mitochondrial organization | 0.036 | 0.62 | 2-oxoglutarate dehydrogenase | Achilles, pan-cancer | TCA cycle,  regulates HIF1α |
|  |  | 0.002 | 0.22 |  | CERES,  pan-cancer |  |
|  |  | 0.038 | 1.07 |  | Marcotte et al,  pan-cancer |  |
| *SLC25A28* | Mitochondrial organization | 0.041 | 0.59 | Mitoferrin-2 | Marcotte et al, pan-cancer | Mitochondrial iron transport |
|  |  | 0.042 | 0.21 |  | CERES,  Breast |  |
| *ISCU* | Mitochondrial organization | 0.023 | 1.78 | Iron-sulfur cluster assembly enzyme | Marcotte et al,  pan-cancer | [Fe-S] cluster synthesis |
| *LIAS* | Mitochondrial organization | 0.0003 | 1.63 | Lipoic acid synthase | Marcotte et al, pan-cancer | Fe_4_S_4_-cluster containing protein, lipoate synthesis, regulates HIF1α |
|  |  | 0.009 | 1.74 |  | Marcotte et al, breast |  |
|  |  | 0.041 | 0.18 |  | CERES, pan-cancer |  |
| *PLOD1* | Protein modification | 0.045 | 0.41 | Procollagen-lysine,2-oxoglutarate 5-dioxygenase 1 | Marcotte, pan-cancer | Iron binding protein, regulates collagen synthesis, cross-linking, and deposition |
|  |  | 0.006 | 0.53 |  | Marcotte, breast |  |
|  |  | 0.042 | 0.07 |  | CERES, breast |  |
|  |  | 0.013 | 0.24 |  | Ataris, pan-cancer |  |
|  |  | 0.043 | 0.41 |  | DRIVE -Ataris,  Breast |  |
|  |  | 0.010 | 0.21 |  | DRIVE -RSA,  pan-cancer |  |
| *BOLA2* | Unknown | 0.035 | 0.10 | BolA-like protein 2 | CERES, pan-cancer | [Fe-S] cluster assembly |

**Supplementary file 1B**

**Iron related genes exhibiting LOF interactions with *MEMO1***

| **Genes** | **GO Slim** | **P-value** | **Distance low-high** | **Protein name** | **Database** | **Protein function** |
| --- | --- | --- | --- | --- | --- | --- |
| *SLC11A2* | Apoptosis | 0.024 | -0.07 | DMT1 | CERES, breast | Iron transport |
| *FTH1* | Immune System process | 0.017 | -0.09 | Ferritin H1 | CERES pan-cancer | Iron storage |
| *FXN* | Mitochondrial organization | 0.032 | -0.79 | Frataxin | Marcotte et al, breast | [Fe-S] cluster biogenesis in mitochondria |
| *FBXL5* | Miscellaneous | 0.038 | -0.57 | F-box/LRR-repeat protein 5 | Marcotte et al, pan-cancer | regulates iron homeostasis |
| *ISCA2* | Mitochondrial organization | 0.038 | -0.39 | [Fe-S] cluster assembly 2 homolog | Marcotte et al, pan-cancer | [Fe-S] cluster biogenesis in mitochondria |
| *NUBPL* | DDR pathways & NA metabolism | 0.013 | -0.69 | Iron-sulfur protein NUBPL | Marcotte et al, pan-cancer | [Fe-S] cluster biogenesis in mitochondria |
| *NCOA4* | Signal transduction | 0.036 | -0.50 | Nuclear receptor coactivator 4 | Achilles, pan-cancer | Mediates ferritinophagy, androgen receptor co-activator, peroxisome (PPAR) receptor co-activator |
|  |  | 0.024 | -0.31 |  | DRIVE-RSA, pan-cancer |  |
|  |  | 0.043 | -1.24 |  | DRIVE-RSA, breast |  |

**Supplementary file 1C**

**Genes involved in ferroptosis and exhibiting GOF or LOF interactions (highlighted in light blue) with *MEMO1***

| **Genes** | **GO Slim** | **P-value** | **Distance low-high** | **Protein name** | **Database** | **Protein function** |
| --- | --- | --- | --- | --- | --- | --- |
| *TFR2* | Ion regulation | 0.032 | 0.06 | Transferrin receptor 2 | CERES, breast | Iron transport |
| *FTH1* | Immune System process | 0.037 | 0.19 | Ferritin H1 | DRIVE -RSA, pan-cancer | Cellular iron storage |
| *ACO1* | Metabolism | 0.019 | 0.16 | Apo-form: Iron-response protein (IRP); holo-form: Aconitase | DRIVE-Ataris, pan-cancer | Iron binding protein, aconitase/IRP |
| *VDAC1* | Ion Regulation | 0.0486 | 0.693 | Voltage-dependent iron channel 1 | Marcotte, pan-cancer | Transport of small hydrophilic molecules; regulation of cell volume and apoptosis |
| *VDAC3* | Ion regulation | 0.032 | 0.452 | Voltage-dependent iron channel 3 | Marcotte breast | Transport of small hydrophilic molecules; regulation of cell volume and apoptosis |
| *SLC25A28* | Mitochondrial organization | 0.041 | 0.59 | Mitoferrin-2 | Marcotte, pan-cancer | Mitochondrial iron transport |
|  |  | 0.042 | 0.21 |  | CERES,  breast |  |
| *ACSL4* | Metabolism | 0.040 | 0.248 | Acyl-CoA Synthetase Long Chain Family Member 4) | Achilles pan-cancer | Lipid metabolism |
| *SIRT3* | unknown | 0.005 | 0.839 | Sirtuin-3 | Marcotte pan-cancer | NAD-dependent deacetylase |
|  |  | 0.042 | 0.554 |  | Marcotte breast |  |
|  |  | 0.009 | 0.539 |  | Achilles pan-cancer |  |
| *SCO2* | Mitochondrial organization | 0.001 | 0.149 | Sco2 | CERES pan-cancer | Mitochondrial copper chaperone |
| *ALOXE3* | metabolism | 0.030 | 0.063 | Hydroperoxide isomerase ALOXE3 | CERES pan-cancer | Non-Heme iron-containing lipoxygenase |
| *PTGS2* | *Intracellular Protein Traffic* | 0.042 | 0.038 | prostaglandin-endoperoxide synthase 2 | CERES breast | Prostanoid biosynthesis |
| *FXN* | Mitochondrial organization | 0.032 | 0.787 | frataxin | Marcotte breast | [Fe-S] cluster biogenesis in mitochondria |
| *CS* | Metabolism | 0.048 | -0.236 | Citrate synthase | Ataris pan-cancer | TCA cycle |
| *ACSL4* | Metabolism | 0.042 | -0.418 | Acyl-CoA Synthetase Long Chain Family Member 4) | Marcotte breast | Lipid metabolism |
| *SCP2* | Cell cycle and Mitosis | 0.024 | -0.651 | sterol carrier protein 2 | Marcotte breast | Intracellular lipid circulation and metabolism |
| *GLS2* | Mitochondrial organization | 0.013 | 0.129 | Glutaminase 2 | CERES breast | Glutamine catabolism, mitochondrial respiration |
| *SCO2* | Mitochondrial organization | 0.042 | 0.744 | Sco2 | Marcotte breast | Mitochondrial copper chaperone |
|  |  | 0.022 | -1.32 |  | Achilles breast |  |
| *FH* | Mitochondrial organization | 0.014 | -0.328 | Fumarate hydratase | Atares pan-cancer | TCA cycle |
| *ALOX12* | Metabolism | 0.044 | 0.049 | arachidonate lipoxygenase | CERES pan-cancer | Lipid peroxidation |

**Supplementary file 1D**

**Structure determination statistics for MEMO1-metal complexes and the C244S mutant**

|  | MEMO1 WT – Fe^2+^ | MEMO1 WT – Cu | MEMO1 C244S - Cu |
| --- | --- | --- | --- |
| Wavelength | 1.03320 | 0.97933 | 0.98011 |
| Resolution range | 47.82 – 2.15  (2.23-2.15) | 42.91- 2.55  (2.64 – 2.55) | 42.1 – 1.75  (1.81 – 1.75) |
| Space group | *P* 2_1_2_1_2 | *P* 2_1_2_1_2 | *P* 2_1_2_1_2 |
| Unit cell (Å) | *a* = 139.64  *b* = 88.86  *c* = 97.36 | *a* = 140.63  *b* = 87.16  *c* = 98.59 | *a* = 140.00  *b* = 89.52  *c* = 97.54 |
| Reflection measured | 880,581 (85,505) | 597,689 (76,841) | 1,834,535 (291,514) |
| Unique reflections | 66,565 (6570) | 40,211 (3967) | 123,799 (20,426) |
| Multiplicity | 13.2 (13.0) | 14.86 (19.37) | 14.82 (14.27) |
| Completeness (%) | 99.9 (99.91) | 99.82 (99.87) | 99.86 (99.68) |
| Mean I/sigma (I) | 19.14 (5.88) | 16.56 (3.13) | 27.83 (3.2) |
| Wilson B-factor | 22.78 | 35.23 | 18.82 |
| R-merge | 0.1351 (0.5015) | 0.186 (1.09) | 0.088 (1.05) |
| CC 1/2 | 0.998 (0.968) | 0.998 (0.886) | 1.0 (0.893) |
| Reflections used for refinement | 66,518 (6564) | 40,172 (3962) | 123,704 (12, 178) |
| R-work | 0.16 (0.20) | 0.19 (0.28) | 0.16 (0.26) |
| R-free | 0.20 (0.25) | 0.25 (0.32) | 0.19 (0.30) |
| Non-hydrogen atoms | 10,319 | 9286 | 11239 |
| Protein residues | 1178 | 1171 | 1176 |
| RMS (bonds) | 0.006 | 0.003 | 0.009 |
| RMS (angles) | 0.83 | 0.51 | 0.94 |
| Ramachandran plot  Favored (%) | 98.12 | 97.51 | 98.37 |
| Allowed (%) | 1.88 | 2.49 | 1.63 |
| Outliers (%) | 0 | 0 | 0 |
| Rotamer outliers (%) | 1.2 | 3.94 | 1.99 |
| Clashscore | 3.46 | 3.33 | 2.46 |
| Average B-factor | 26.46 | 44.79 | 22.70 |
| No of TLS groups | 4 | 4 | 4 |
| PDB code | 7KQ8 | 7L5C | 7M8H |
